# PD-1 inhibitor therapy causes multisystem immune adverse reactions: a case report and literature review

**DOI:** 10.3389/fonc.2022.961266

**Published:** 2022-09-02

**Authors:** Na Yin, Xiangliang Liu, Xiaojun Ye, Wei Song, Jin Lu, Xiao Chen

**Affiliations:** Cancer Center, The First Hospital of Jilin University, Changchun, China

**Keywords:** immunotherapy, lung cancer, immune checkpoint inhibitor, myocarditis, myositis, thrombocytopenia, IL-6

## Abstract

Immune checkpoint inhibitors(ICIs), including cytotoxic T-lymphocyte antigen 4 (anti-CTLA-4), programmed cell death protein 1 and its ligand (PD-1/PD-L1) inhibitors, have been shown to have antitumor activity in various solid tumors. Their mechanism of action is to selectively restore and normalize the body’s immune reponses by disrupting the immunosuppressive signals mediated by PD-1, PD-L1 and CTLA-4 in the tumor microenvironment. With the increase in clinical applications of ICIs, reports of immune-related adverse events (irAEs) have also increased. This article reports a case of a lung cancer patient who developed multisystemic adverse effects after PD-1 inhibitor application: myocarditis, myositis and thrombocytopenia, and analyzes the role of Interleukin 6(IL-6)in the management of irAEs. Despite the patient’s eventual discontinuation of antitumor therapy due to severe irAEs, a significant and durable therapeutic response was observed.

## Introduction

ICI enhances the anti-tumor activity of the host immune system by blocking checkpoint molecules. The results of clinical studies have shown that ICI has clear effects in the treatment of melanoma and advanced non-small cell lung cancer and can also be applied in the treatment of malignant tumors such as breast, head and neck, gastric, uroepithelial, and lymphoma (1, 2). With the increase in clinical use, ICIs have developed immune-related adverse reactions (irAEs) (3, 4), mainly in vital organs such as the skin, gastrointestinal tract, endocrine glands, liver and lungs, and have potential effects on other organs and tissues. Glucocorticoids are the first line of treatment for irAE. If steroid fails, second-line treatment is considered. The main drugs are inhibitors of T-cell immunity, including mycophenolate mofetil (MMF), azathioprine (AZA), anti-human thymocyte immunoglobulin (ATG) and tacrolimus. Immunoglobulins, plasma replacement and new biologics such as infliximab have also been used to suppress immunity. Esfahani’s team noted that a comprehensive assessment of the histological features of the organs involved in irAE, obtaining peripheral blood flow cytology and measuring autoantibody levels and cytokines is needed before treatment. They suggest a more refined classification and treatment of irAE based on individualized features (5).

Among cardiac adverse reactions, myocarditis has a low incidence but can be life-threatening, with a mortality rate of 25% to 50% (6, 7). Here, we report a case of ICI-induced multisystemic adverse effects after the treatment of anti-PD-1 therapy for lung adenocarcinoma in which immune-associated myocarditis can be life-threatening. A 67-year-old gentleman with lung adenocarcinoma was given 5 cycles of chemotherapy combined with immunotherapy as second-line treatment after disease progression from first-line chemotherapy. The patient mainly complained about persistent weakness and myalgia followed by chest pain, dyspnea, and markedly elevated laboratory parameters such as troponin and cytokines. He was diagnosed as ICI-induced myocarditis overlapping with myositis and thrombocytopenia. Then, his symptoms were resolved after prompt therapy with high-dose steroids. Our report aims to raise awareness of the early prediction, early intervention and correct treatments in ICIs-induced rare side effects during the immune checkpoint blockade treatments of common tumor. It also initially analyzes the role of Interleukin 6(IL-6 ) in the management of irAEs and the relationship between irAE episodes and ICI outcomes, providing clinical evidence for real-world research.

## Case presentation

**Figure 1:**
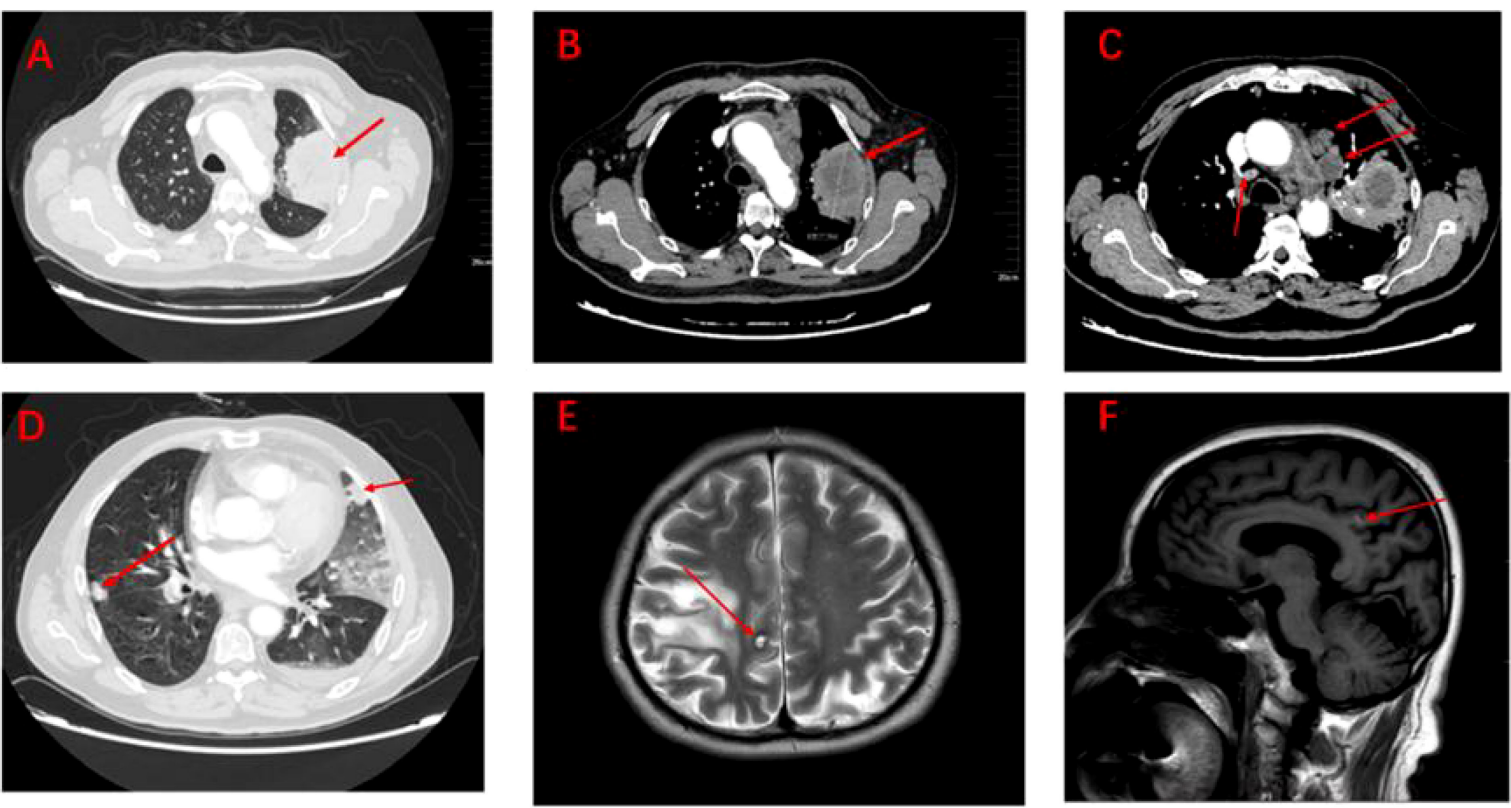
Imaging of tumour lesions at baseline **(A–F)**. **(A)** Pulmonary lesions under the lung window. **(B)** Pulmonary lesions under the mediastinum window (measuring 4.8cm x 4.6cm x 6.1cm). **(C)** Lymph node metastatic lesions under the mediastinum window. **(D)** Metastatic lesions in both lungs under the lung window. **(E)** Brain metastases under the coronal plane. **(F)** Brain metastases under the sagittal plane.

**Figure 2:**
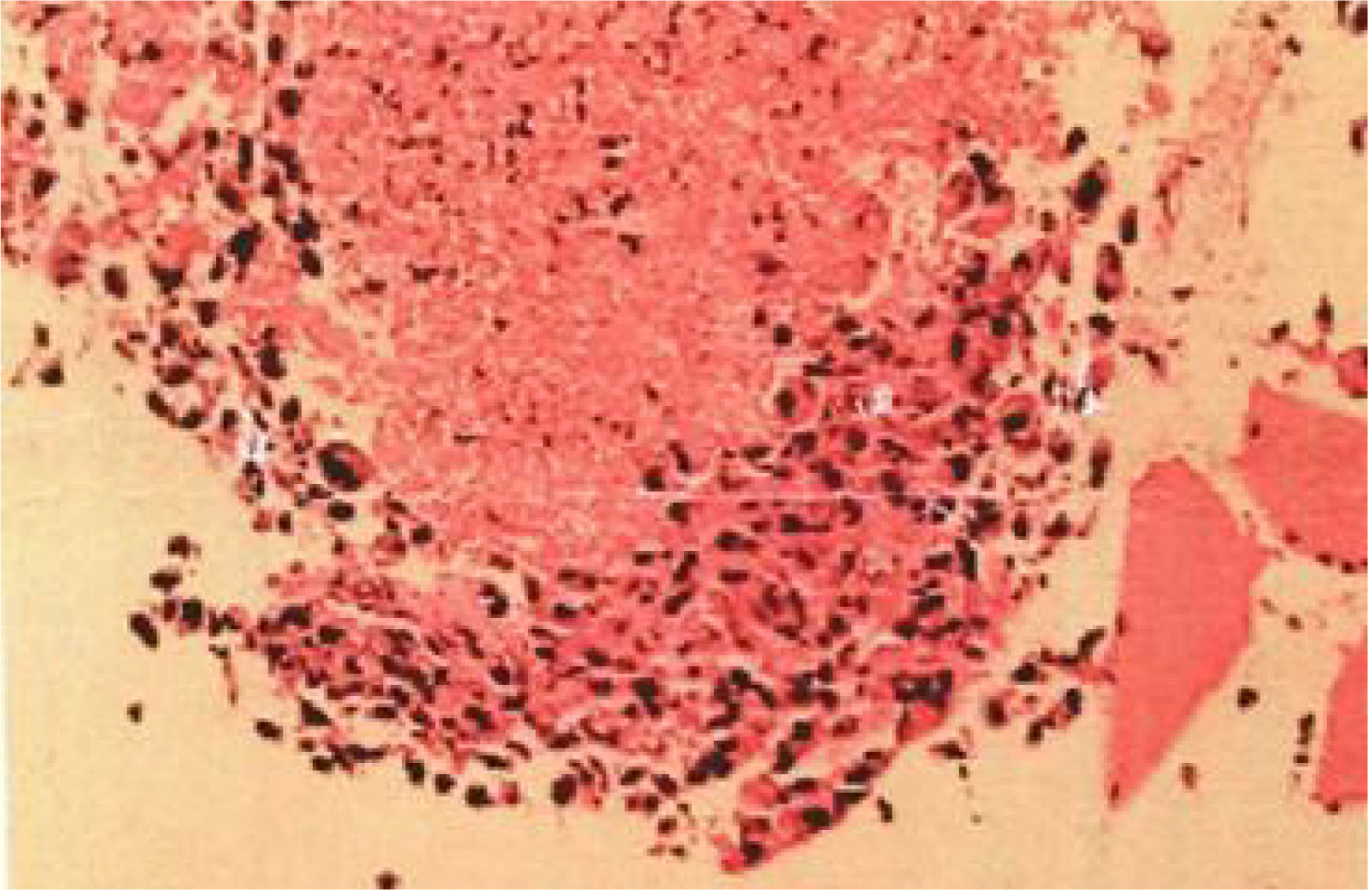
Lung tissue sections stained with hematoxylin-e.

**Figure 3:**
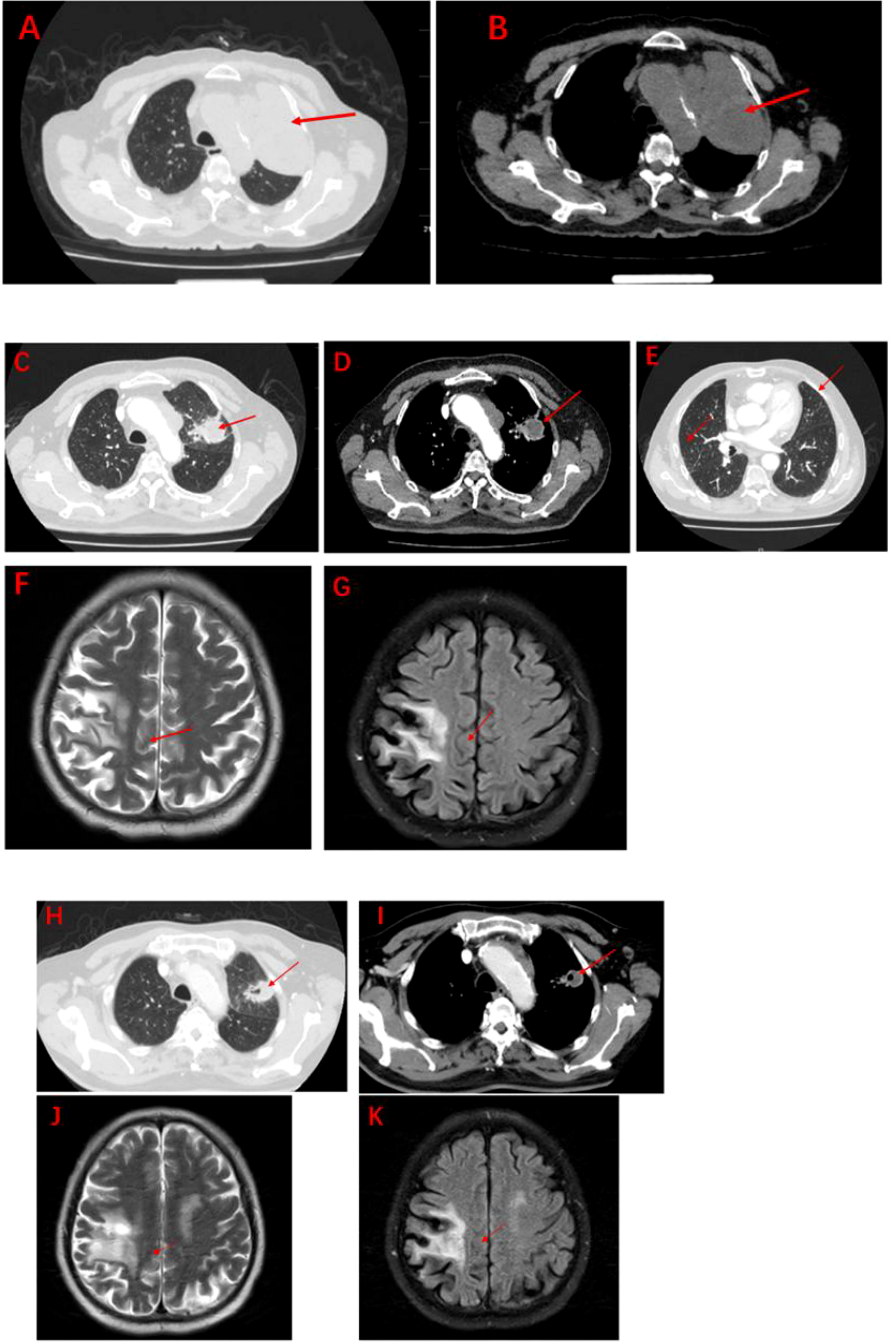
Imaging after anti-tumour therapy **(A–K)**. **(A, B):** Significant increase in lung lesions and bilateral lung metastases after 1 course of pemetrexed in combination with carboplatin (measuring 5.5cm x5.7cm x7.1cm). **(C-E):** The second line of treatment was “albumin paclitaxel + nedaplatin + PD-1 inhibitor (Sintilimab)”, and the lung lesions and metastases were significantly reduced after 2 courses (measuring 3.3cm x2.8cm x4.6cm). The efficacy assessment reached PR. **(F, G)**: Significant reduction of brain metastases after 2 courses of Sintilimab in combination with chemotherapy. **(H, I):** The lung lesions continue to shrink after 4 courses of Sintilimab in combination with chemotherapy (measuring 2.8cm x 2.4cm x 3.9cm). **(J, K):** Brain metastases barely detectable after 4 courses of Sintilimab in combination with chemotherapy.

**Figure 4:**
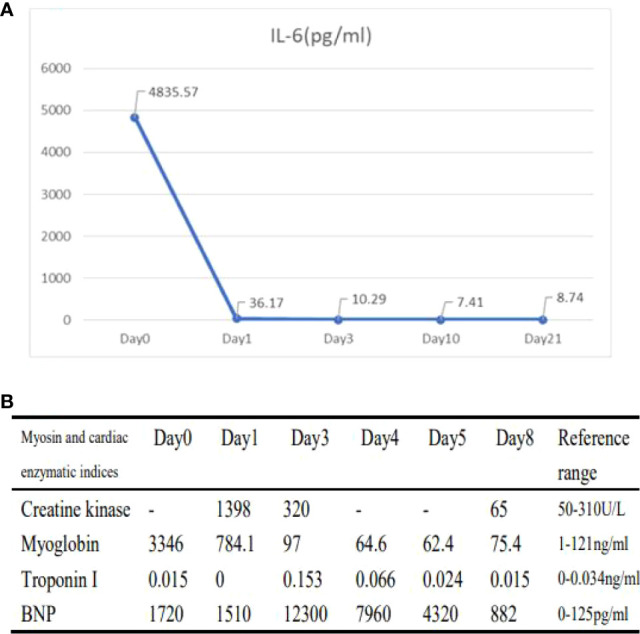
Changes in laboratory indicators during the onset of immune-related toxicity in patients **(A, B)**. **(A)**: Changes in IL-6 (Day0 represents the level after 0 days of steroid treatment; Day1 represents the level after 1 days of steroid treatment; Day3 represents the level after 3 days of steroid treatment; Day10 represents the level after 10 days of steroid treatment; Day21 represents the level after 21 days of steroid treatment.) **(B)** Changes in cardiac parameters (Day0 represents levels after 0 day of steroid treatment; Day1 represents levels after 1 day of steroid treatment; Day3 represents levels after 3 days of steroid treatment; Day4 represents levels after 4 days of steroid treatment; Day5 represents levels after 5 days of steroid treatment; Day8 represents levels after 8 days of steroid treatment).

**Figure 5:**
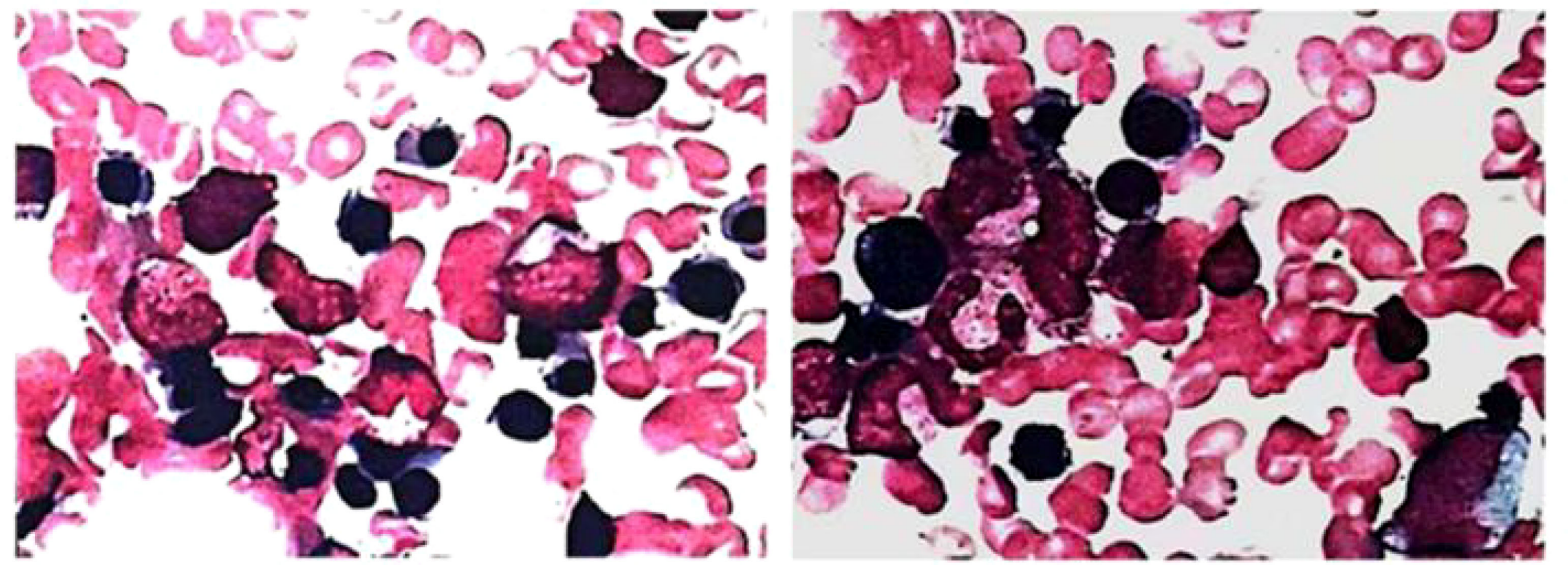
Bone marrow evaluation showed poor maturation of megakaryocytes.

**Figure 6:**
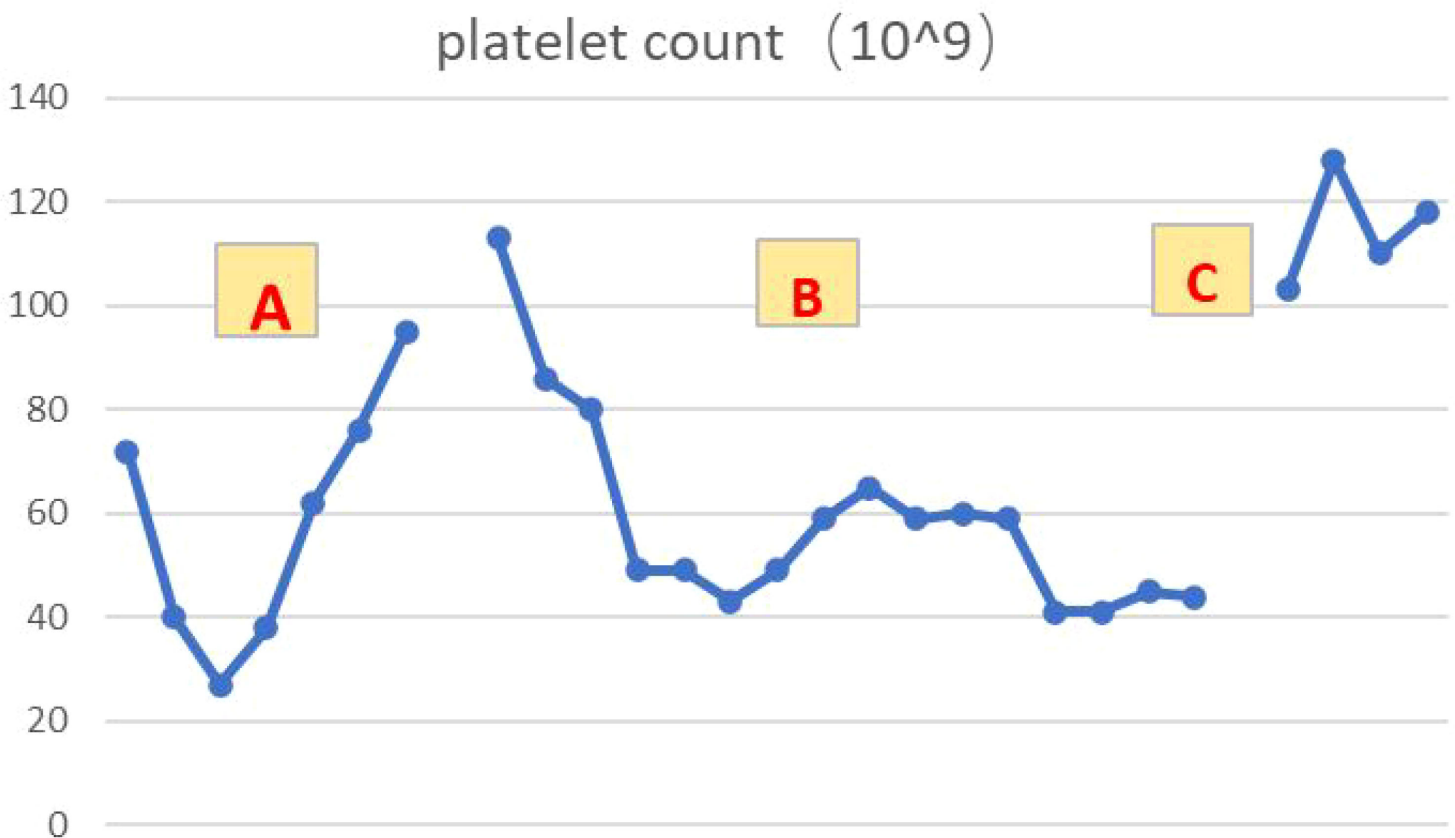
Platelet changes during the course of the disease **(A–C)**. **(A):** Reduced platelet levels after 5 courses of chemotherapy combined with immunotherapy, with gradual recovery after symptomatic treatment. **(B):** Platelet levels decreased again after 1 course of immune consolidation therapy. **(C):** After discharge from hospital, oral steroids were administered and platelets recovered and maintained in the normal range.

**Figure 7:**
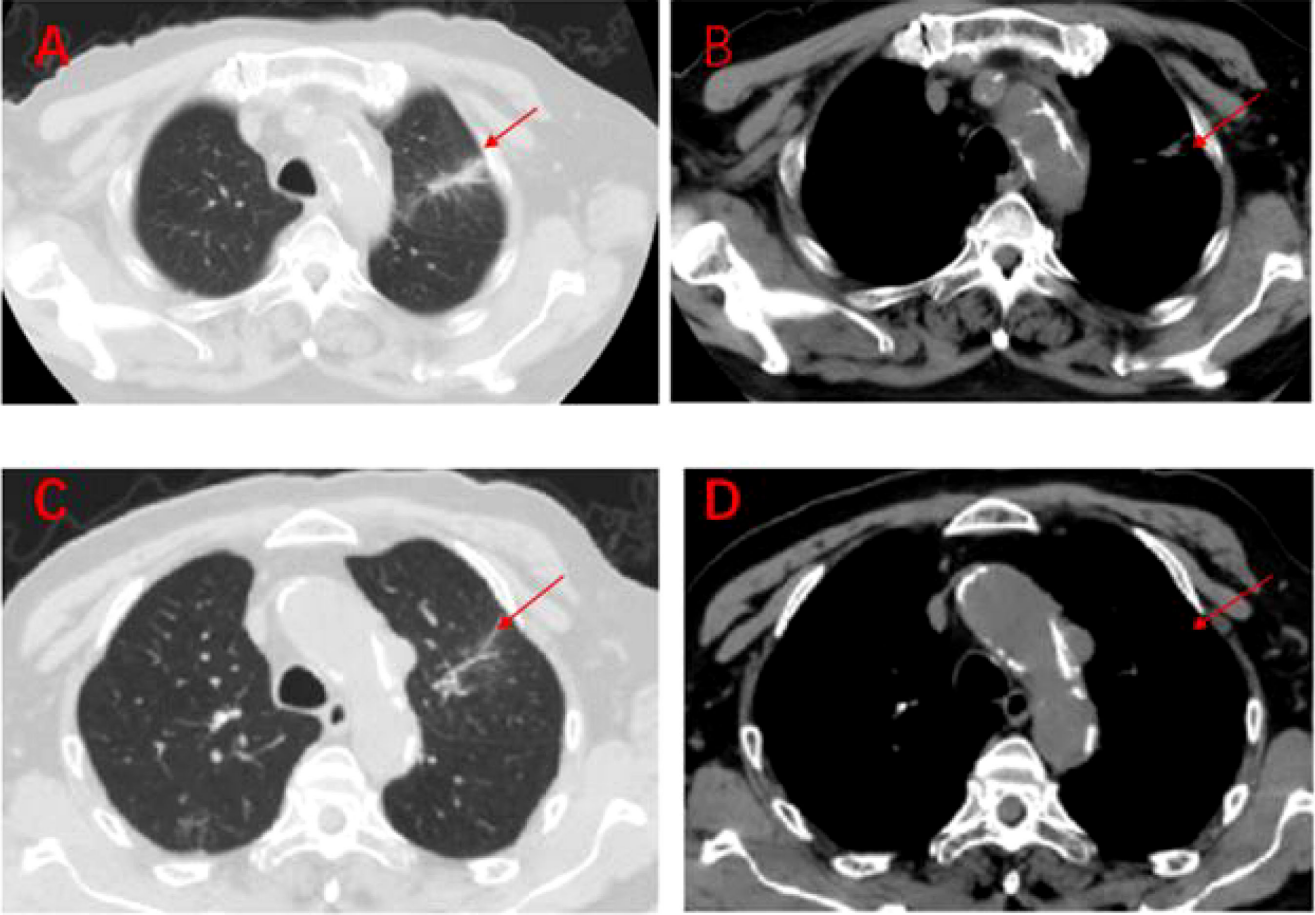
Regular imaging at follow-up, tumour lesions continue to shrink, efficacy maintained at PR **(A–D)**. **(A, B)**: On 7 March 2020, a lung CT scan revealed almost no tumour lesions. **(C, D)**: On 3 October 2020, a lung CT scan revealed almost no tumour lesions.

**Figure 8:**
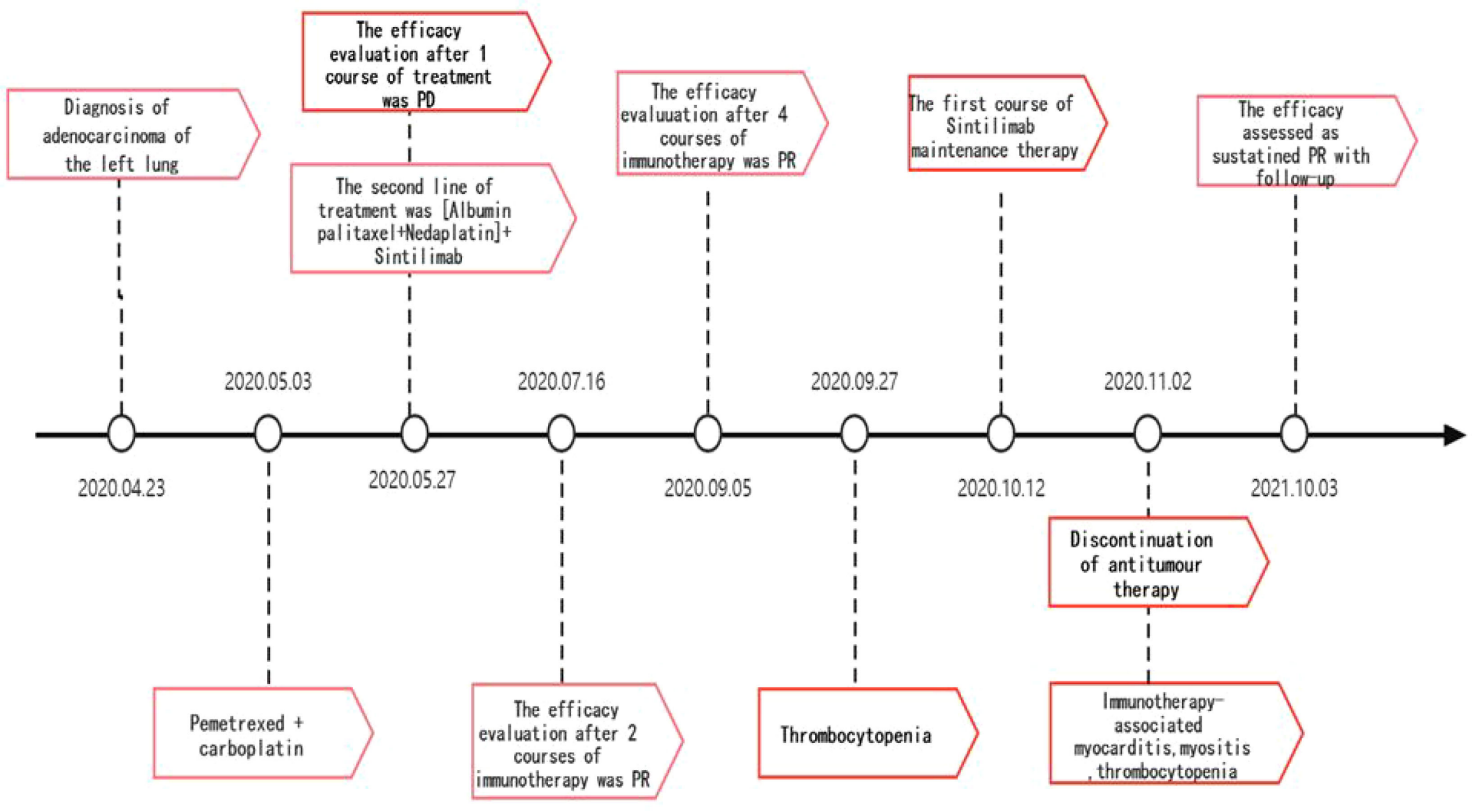
A summary of a clinical course.

The patient, a 67-year-old male, presented to our hospital in April 2020 with “hoarseness with hemoptysis”. He had no history of hypertension, diabetes mellitus, coronary heart disease, asthma, liver disease or other underlying diseases. He also had no family history of tumours. After admission, the patient underwent a lung computed tomography (CT) examination, which revealed a mass-like high-density shadow of about 4.8x4.6x6.1 cm in the upper lobe of the left lung (**Figures 1A, B**), considering peripheral lung cancer; enlarged lymph node shadow in the mediastinum (**Figure 1C**), considering metastatic cancer; nodular high-density shadow in both lungs (**Figure 1D**), considering metastatic cancer. Pathological findings on lung puncture biopsy suggested hypofractionated adenocarcinoma with immunohistochemical results showing CK5/6 (partial +), CK7 (+), Ki-67 (+80%), Napsin A (-), P40 (-), Vimentin (+), CK-pan (+), TTF-1 (+) (**Figure 2**). A head magnetic resonance imaging (MRI) performed for a systematic evaluation suggested abnormal signal in the right parietal lobe (**Figures 1E, F**), and metastases were considered. No evidence of metastases was found on abdominal CT and bone scan. Pathologic staging was defined according to the American Joint Committee on Cancer (AJCC) TNM staging system, 8th edition, and combined with imaging and pathologic findings, the clinical diagnosis of this patient was left lung adenocarcinoma with bilateral lung and brain metastases (cT4N3M1b stage IV). Genetic testing suggested a positive K-RAS gene. After one cycle of chemotherapy with “pemetrexed + carboplatin”, a repeat lung CT showed that the lung lesions, lymph nodes and bilateral lung metastases were larger than before (**Figures 3A** **, B**). The second line of treatment was “albumin paclitaxel + nedaplatin + PD-1 inhibitor (Sintilimab)”, and the lung lesions (**Figures 3C** **, D**) and metastases (**Figures 3E** **–G**) were significantly reduced after 2 cycles. The efficacy assessment reached PR (partial response), and chemotherapy combined with immunotherapy was continued for 3 cycles. During the treatment, regular monitoring of routine blood, liver and kidney function, immunological indexes (including cardiac enzymes, thyroid function, pituitary function, etc.) and electrocardiogram and cardiac ultrasound did not reveal any significant abnormalities. The efficacy was assessed as maintenance PR (**Figures 3H–K**). On September 26, 2020, the patient was admitted to the hospital with “peripheral discomfort with marked malaise and nausea”, and laboratory tests showed a decrease in platelets with a minimum value of 27X10^9/L. After symptomatic treatment (thrombopoietin 15000 IU, once per day, subcutaneous injection), platelets were restored to normal, and PD-1 inhibitor (sintilimab) was administered as maintenance therapy. On November 1, 2020, the patient was readmitted to the hospital with “increased malaise with myalgia”. In the afternoon of the second day of admission, the patient developed fever with a maximum temperature of 40.1°C, respiratory distress, increased heart rate, decreased oxygen saturation, and drowsiness, accompanied by a decrease in blood pressure (minimum value of 74/43 mmHg). Laboratory tests showed creatine kinase (CK) 1398 U/L (normal range: 50-310 U/L), myoglobin 3346 ng/ml (normal range: 1-121 ng/ml), troponin I 0.153 ng/ml (normal range: 0-0.034 ng/ml), brain natriuretic peptide (BNP) 12300 pg/ml (0- 125 pg/ml), which were significantly elevated, serum IL-6 was 4835.57 pg/ml and IL-10 was 122.18 pg/ml. Electrocardiogram suggested tachycardia, the rightward shift of the cardiac axis, and ischemic-type changes in the ST segment of leads II and V4. Based on medical history and auxiliary examinations, coronary diseases causing ST-segment ischemic manifestations were ruled out. ICI-mediated myocarditis and myositis was highly considered. Based on the above information, steroids were given to the patient according to the changes in troponins, myocardial enzymes and symptoms (methylprednisolone ivvp. 120mg q12h for 10 days, 120mg qd for 3 days, 80mg qd for 4 days, 60mg qd for 6 days, 40mg qd for 5 days). Gradually, the patient’s vital signs were relatively stable. Serum IL-6 decreased to 36.17 pg/ml and IL-10 decreased to 8.38 pg/ml. Creatine kinase, myoglobin, troponin and BNP gradually returned to normal (changes in laboratory indicators are shown in the **Figures 4A** **, B**). Furthermore, on day 3 of hospitalization, the patient again developed thrombocytopenia with a minimum value of 41X10^9/L. Bone marrow evaluation (**Figure 5**) showed poor maturation of megakaryocytes, and immune-associated thrombocytopenia was considered. And evidence of metastatic cancer invasion was not present. After excluding chemotherapy, infectious etiology, or other drug-induced thrombocytopenia, we considered a diagnosis of immune thrombocytopenia induced by sintilimab. Patient continued to receive steroids. After discharge, oral prednisone was administered and gradually tapered. His platelets returned to normal on December 12,2020 (changes in platelet levels are shown in **Figure 6**). No further immune-related adverse events occurred. Regular imaging examinations were performed, the tumor lesions continued to shrink, and the efficacy maintained at PR (**Figure 7**). At present, the patient is still under regular follow-up. The administration of immune-related toxicity and the effect of treatment are shown on the **Table 1**. The clinical course of this patient is summarized in **Figure 8**.

**Table 1:** The administration of immune-related toxicity and the effect of treatment.

IRAE	Treatment	Treatment effects
Myocarditis	**During hospitalization:** Methylprednisolone ivvp. 120mg q12h for 10 days, 120mg qd for 3 days, 80mg qd for 4 days, 60mg qd for 6 days, 40mg qd for 5 days. **After discharge:** oral prednisone was administered and gradually tapered.	Gradually, the patient’s vital signs were relatively stable. Serum IL-6 decreased to 36.17 pg/ml and IL-10 decreased to 8.38 pg/ml. Creatine kinase, myoglobin, troponin and BNP gradually returned to normal.
myositis		Weakness and myalgia gradually disappear
thrombocytopenia		Platelets gradually recover and eventually stabilize in the normal range.

## Discussion

### Immune-associated myocarditis

Our understanding of the pathophysiological mechanisms of ICI-induced myocarditis comes from animal studies (8), and data from relevant animal model studies suggest that the PD-1/PD-L1 and CTLA-4 signalling pathways downregulate excessive immune responses in cardiomyocytes and have essential protective effects on the myocardium (9). Early on, it has been shown that CTLA-4 and PD-1 deficiency can cause autoimmune myocarditis (10, 11). Both PD-1-CD4+ T cells and PD-1-CD8+ T cells mediate myocardial injury, and both T cell subsets require PD-1 to maintain their tolerance to myocardium (12). PD-L1, the ligand of PD-1, is expressed in the myocardium of both humans and mice. It was found that genetic deletion of both PD-L1 and PD-L2 and the role of ICI can cause transient myocarditis that eventually progresses to fatal disease, confirming that PD-1 signalling plays a key role in protecting the myocardium from damage by T-lymphocyte immune responses (13). In addition, recent case reports have found autoantibodies detected in patients with ICI-associated myocarditis, suggesting antibody-mediated myocardial injury (14). Future studies need to clarify further the mechanisms of toxicity and associated risk factors in ICI-associated myocarditis.

Clinical symptoms of immune-related cardiovascular toxicity are varied and may present as mild non-specific symptoms such as malaise and weakness. Typical symptoms associated with cardiac diseases such as dyspnea, chest pain, pulmonary edema, bilateral lower extremity edema, cardiac arrhythmia, and acute heart failure may also be present. Other atypical symptoms include myalgia and syncope. There are no uniform diagnostic criteria for immune-associated myocarditis, and the generally accepted gold standard is endomyocardial biopsy or histopathological findings. Still, its invasive nature, risk of cardiac perforation and time-consuming biopsy limit its application. The sensitivity and specificity of cardiac magnetic resonance T1-weighted and T2-weighted images and late gadolinium enhancement for the diagnosis of immune myocarditis are 76% and 96%, respectively (15). Echocardiography may suggest abnormal ventricular wall motion, and electrocardiogram may be positive. However, immune myocarditis often occurs insidiously, progresses relatively rapidly, and most patients presenting to the clinic are more severe, making it difficult to obtain definitive diagnostic evidence. According to guideline recommendations (16), cardiac troponin (cTn) and creatine kinase (CK) can be used to guide the diagnosis and management of ICI-associated myocarditis. Therefore, it is particularly important to advise patients to monitor cardiac enzyme profiles (troponin I or T, CK, CK-MB, and natriuretic peptide) at baseline and periodically during drug administration. However, the clinical value of serum biomarkers (e.g., troponin) for the early detection of ICI-associated myocarditis still needs to be confirmed by further evidence and studies.

The patient had significant chest tightness, palpitations, and precordial discomfort during maintenance treatment with PD-1 inhibitor. Laboratory tests suggested that the cardiac enzyme profile was elevated to 0.153 ng/ml for ultrasensitive troponin I and 12,300 pg/ml for BNP. Electrocardiogram suggested ST-segment ischemic changes, but cardiac ultrasound showed no abnormalities. According to NCCN guidelines, abnormal echoes suggestive of echocardiography without hypotension and cardiac markers >3 times the upper limit of normal were defined as severe ICI cardiovascular toxicity. Heart rate arrhythmia, hemodynamic instability (hypotension/cardiomyopathy), and cardiac markers >3 times the upper limit of average values were defined as life-threatening cardiovascular toxicity (17). Therefore, this patient was diagnosed with ICI-associated myocarditis with reduced blood pressure, classified as severe cardiovascular toxicity and significantly controlled with prompt steroids therapy.

Skeletal and cardiac muscles belong to the same transverse muscle. Some studies (18, 19) have found that anti-transverse muscle antibodies mediate both immune myositis, myocarditis and myasthenia gravis, which may act as biomarkers for these immune-related adverse events. This suggests that autoimmune targets with similar epitopes may exist in cardiac and skeletal muscle. In addition, the PD-1 signaling pathway plays an important role in the autoimmune response of these tissues (12, 20). Usually, the main clinical manifestation of skeletal muscle toxicity after immune checkpoint inhibitor therapy is a weakness with myalgia, characterized by elevated serum creatine kinase and myoglobin, and in severe cases, rhabdomyolysis. In this case, the main manifestation of skeletal muscle toxicity was “weakness with myalgia” before admission to the hospital. After admission, creatine kinase 1398 U/L and myoglobin 3346 U/L were 10 times higher than normal. The patient was considered to have skeletal muscle and cardiac muscle damage, and serum IL-6 was elevated to 4835.57 pg/ml and IL-10 to 122.18 pg/ml, suggesting an immune storm. After steroids treatment, his weakness and myalgia symptoms gradually improved, and creatine kinase and myoglobin levels gradually returned to normal. Therefore, the manifestation of skeletal muscle injury due to ICI may be an early stage of immune-associated myocarditis, and early recognition would be beneficial to improve the patient prognosis.

### Immune-related thrombocytopenia

The accepted mechanisms regarding immune-associated thrombocytopenia are antibody-driven and T-cell-mediated. It has been suggested that activation of CD4+ helper T cells and CD8+ cytotoxic T cells in patients treated with ICIs are involved in the immune response, leading to hematopoietic stem cell injury and inducing immune-associated thrombocytopenia and other hematologic complications (21). A single case of non-small cell lung cancer (NSCLC) with nivolumab reported that nivolumab induces or increases the production of platelet auto-specific Ig antibodies, which leads to impaired platelet maturation and reduced platelet production by bone marrow megakaryocytes (22). Thrombocytopenia is associated with the presence of platelet antibodies, autoantibodies, and thyroglobulin antibodies, and is accompanied by a decrease in the number of helper T cells and regulatory T cells (23). In addition, there is evidence that PD-1, Treg pathways may be involved in the development of immune-related thrombocytopenia. Compared to healthy individuals, peripheral blood T cells of immune thrombocytopenic individuals have lower PD-1 expression, and PD-1 levels are lower in acute thrombocytopenic individuals than in chronic individuals (24). Furthermore, bone marrow biopsy revealed that the bone marrow of immune platelet-depleted individuals expressed lower Treg than healthy individuals, suggesting that platelet decline may be associated with Treg (25). Regardless of the mechanism of occurrence or clinical features, immune thrombocytopenia caused by ICIs has similarities to classical immune thrombocytopenia (26), and interference from infection, tumor progression, and other chemotherapeutic agents used in combination with ICIs needs to be excluded.

In this case, the patient developed thrombocytopenia after 5 cycles of chemotherapy combined with immunotherapy. After symptomatic treatment, the platelets returned to normal, at which point the cause of thrombocytopenia would conventionally be considered to be related to chemotherapy. However, the platelets were again reduced after discontinuing chemotherapy drugs and continuing to PD-1 inhibitors. Further observation, we found that there was an overlap between the time points of thrombocytopenia and IL-6 elevation, a phenomenon that indirectly suggests that cytokines further promote immune disorders during irAEs (27). Bone marrow smear examination suggested poor maturation of megakaryocytes and scattered rare platelets, which were considered secondary alterations. After steroids treatment, platelets recovered and were maintained at normal. In this case, we thought thrombocytopenia as a high probability of hematologic toxicity due to ICI, and IL-6 plays an essential role in immune disorders.

### Role of IL-6 in irAEs

Interleukin-6 (IL-6) is an inflammatory cytokine which has a critical role in the systemic immune system and is associated with various diseases, including cancer (28). IL-6 signaling is complex. At low levels, IL-6 activates anti-inflammatory pathways *via* classic signaling. However, as observed in CRS (Cytokine Release Sydrome), IL-6 at high levels causes proinflammatory effects *via* trans-signaling (29). Common features of the clinical presentation of irAEs were that of a systemic inflammatory response, with an increase in circulating pro-inflammatory cytokines likely triggered by ICI-induced T-cell stimulation (30, 31). Although the pathogenesis of irAEs remains to be clarified, it is hypothesized that irAEs are related to infiltration of activated CD8+ and CD4+ T-cells in target tissues (30) and elevated serum levels of inflammatory cytokines (including IL-6) (31–33). IL-6 promotes tumor progression and metastasis through multiple mechanisms including feed forward activation of oncogenic pathways, inhibition of dendritic cell differentiation, and myeloid-derived suppressor cells augment (34).

CRS may occur after immunotherapy. Studies suggested IL-6, TNF-α, IFN-γ and CRP as monitoring indicators to avoid severe CRS (35, 36). However, the utility of IL-6 as a biomarker for irAE development is largely unknown. IrAEs effect may induce IL-6, especially against the PD-1/PD-L1 axis (37). Literature summarizing a series of cases and studies has found that baseline levels of some cytokines (including IL-6) may be low in patients with irAEs, but changes that rise abruptly after treatment may be associated with irAEs (38). A case report on immune-associated pneuomonitis also tentatively suggested that elevated IL-6 and CRP (C-reactive protein, as downstream molecular product of IL-6) after PD-L1 inhibitors were associated with the development of irAEs in non-small cell lung cancer (NSCLC) and that IL-6 could be one of the potential mediators of irAEs in NSCLC patients treated with ICIs (39). A case report (37) found that elevated serum IL-6 and CRP were proportional to the severity of ICI-associated colitis, and after receiving steroids, their decreased levels were proportional to the degree of remission of colitis, and the results suggest that IL-6 and CRP may be biomarkers for the diagnosis and prediction of irAEs. Two retrospective case studies exploring the efficacy of tocilizumab(IL-6 receptor antagonist)showed that tocilizumab may be a therapeutic strategy for the management of steroid refractory irAEs secondary to immune checkpoint blockade. Moreover, in most cases in both studies, biomarkers of the inflammatory process (IL-6 or CRP levels) decreased rapidly after tocilizumab treatment with clinical improvement and symptom relief, demonstrating the clinical significance of IL-6 in the pathogenesis and management of these events (40, 41).

In this case, the cytokine IL-6 rose to thousands of times its normal level while multiple irAEs were present, which together indicates T cell hyperactivity. After steroids, IL-6 gradually decreased to near normal. Thus, this case also suggests that IL-6 may be a sensitive indicator of specific immune-related adverse reactions, but its sensitivity still needs to be investigated by a large amount of data and experiments. Therefore, routine monitoring of IL-6 and CRP in patients treated with ICIs would be helpful in predicting the clinical course of irAEs.

### Immunotherapeutic efficacy and immune-related adverse events

Immunotherapy has changed the therapeutic landscape of oncology, and the exploration of effective biomarkers to identify patients most likely to benefit from ICI is one of the hot topics in oncology. To date, predictive biomarker studies have focused on pre-treatment tumor characteristics such as microsatellite instability status, PD-L1 expression and tumor mutational load. Clinical biomarkers in treatment have been less studied. A growing number of studies have found a correlation between the incidence of irAEs and treatment response to ICI (42–46). However, the mechanism between irAE appearance and antitumor effect is not yet apparent. Under molecular mechanisms, irAE may be triggered by a common antigen expressed by tumor and inflamed organ (19, 47–49). Unleashed T cells produce toxicity and response by binding to T cell receptor in target tissues. Besides, study suggests gut microbiome may be a complementary explanation for the relationship between irAE and immune efficacy (50, 51). Gut microbes are diverse and complex in composition, so gut microbiome mechanism still need extensive prospective studies to explore. Unlike the two views above, other studies suggest that tissues which develop autoimmune toxicity after ICIs may express organ-specific antigen independent of antitumor response, i.e., such organ-specific antigen mag be pre-existing (52). Onset of irAE may predict response to PD-1 and PD-L1 antibodies, this correlation has been demonstrated in various advanced malignancies, including melanoma (42, 44), NSCLC (45, 46), and gastrointestinal tumor (53), etc. Most of these studies concluded that patients experiencing irAE show significant improvements in progression-free survival, overall survival, or overall remission rates. Studies on the relationship between immunotoxicity and efficacy of CTLA-4 antibody mainly focus on melanoma. Some studies affirm the predictive role of irAE in response of CTLA-4 antibody (43), but others also questione this hypothesis (54).

Key questions regarding the association between irAE onset and ICI efficacy remain. The primary concerns involve whether irAE site, quantity, severity, timing of onset and management influence ICI efficacy. Most studies favor the perspective that patients cutaneous or endocrine (e.g., thyroiditis) irAE exhibit better PFS, OS benefit (44–46, 55, 56). This correlation may stem from the hypothesis that tumor cells express the same antigens as target organ (57). The number of irAEs may influence irAE versus ICI efficacy. The group experiencing ≥2 irAEs present an unprecedented OS benefit compared to the Nivolumab treatment group experiencing one irAE, which indicate that multiple immunotoxicity can reflect a sustained antitumor response (46). IrAE severity is positively correlated with immune efficacy, deriving from higher T-cell activity and stronger immunosuppressive effects in severe irAE (58). Steroids are usually applied against irAE, but steroids are known to be immune-suppressive. The study found that treatment effect compared with placebo after an irAE onset and after day 30 of steroid use appeared to be lower than the effect after an irAE onset and without steroid or by day 30 of steroid use (42). However, other studies suggested that patients on low-dose steroid show a better survival benefit and that high-dose steroid for irAE may diminish ICI efficacy (59). Another study found two lung cancer patients treated with high-dose steroids after developing autoimmune colitis, yet a sustained tumor remission was still observed (43). It is surprising to note that eliminating the autoimmune adverse effects of anti–CTLA-4 with steroids did not seem to interfere with antitumor activity.

But, in short, a full understanding of the true impact of irAE characteristics on ICI efficacy still needs to be demonstrated in larger prospective studies. The patients in this report achieved a PFS of more than 1 year after the occurrence of irAEs, discontinuation of immunotherapeutic agents, and no further antitumor therapy. In addition, the decision to restart immunotherapy in patients who develop severe ICI-related toxicity needs to be made by a clinical multidisciplinary team after considering the risks and benefits of treatment.

With the boom in immunotherapy, preclinical models related to immunotherapy are at the forefront of the medical field. Animal and *in vitro* models have been used for cancer pathogenesis, signaling pathways, therapeutic screening and translational applications (60–62). Given that, many groups are developing elegant and specific preclinical models to examine irAEs. One study used a syngeneic murine Head and neck squamous cell carcinoma (HNSCC) cell panel to accurately recapitulate the tumor immune microenvironment (TIME)and further explore new immune therapeutic options (63). Using a mouse model of HNSCC, Gilardi M team developed a novel, local delivery strategy based upon an array of soluble microneedles (MN). Local-MN delivery of anti-CTLA-4 *in vivo* can protect animals from irAEs observed, but this process relies on CD8 T cells and conventional dendritic cell type 1 (cDC1) (64). Additionally, the 3D culture models of the tumorpromoting microenvironment *in vitro* will contribute to a comprehensive understanding of the mechanisms of malignant metastasis *in vivo* and facilitate the development of novel anti-tumour drugs (65, 66). The microfluidic technology-based method for lung cancer cell-lines categorization is an efficient and promising model for lung cancer differential diagnosis (67). Microfluidic vascular *in vitro* models are considered an ideal model to replicate and mimic the *in vivo* metastatic progression (68). The complexity of the TIME constitutes a major mechanism of resistance to immunotherapy. Immunotherapy-related toxicity is a major concern. The analysis and modeling of the complexity of the microenvironment should receive more attention in the field of immuno-oncology. Therefore, given the results obtained in the above studies, future work will extend the framework to predict the occurrence of immunotherapy resistance and irAE.

## Conclusion

In summary, clinical vigilance should be increased for rare fatal immunotoxicity caused by PD-1 inhibitors. In this paper, we summarize the diagnosis, treatment, and regression of a case of severe immune myocarditis with myositis and thrombocytopenia caused by PD-1 inhibitors, suggesting the importance of early diagnosis and intervention. This case also provides a preliminary analysis of the predictive value of IL-6 for irAE, as well as real-world clinical evidence for the correlation between irAE and immune efficacy.

## Data availability statement

The original contributions presented in the study are included in the article/supplementary material. Further inquiries can be directed to the corresponding author.

## Author contributions

NY and XL contributed equally to this work. NY provided case information and contributed to data analysis and manuscript writing. XL drafted the manuscript. XY, WS, and JL performed the clinical management of the patient. XC revised the article critically for important intellectual content and contributed to the project development. All authors contributed to the article and approved the submitted version.

## Acknowledgments

We thank all the people who have been helpful to this manuscript.

## Conflict of interest

The authors declare that the research was conducted in the absence of any commercial or financial relationships that could be construed as a potential conflict of interest.

## Publisher’s note

All claims expressed in this article are solely those of the authors and do not necessarily represent those of their affiliated organizations, or those of the publisher, the editors and the reviewers. Any product that may be evaluated in this article, or claim that may be made by its manufacturer, is not guaranteed or endorsed by the publisher.
